# Linking the Epigenome to the Genome: Correlation of Different Features to DNA Methylation of CpG Islands

**DOI:** 10.1371/journal.pone.0035327

**Published:** 2012-04-30

**Authors:** Clemens Wrzodek, Finja Büchel, Georg Hinselmann, Johannes Eichner, Florian Mittag, Andreas Zell

**Affiliations:** Center for Bioinformatics Tübingen (ZBIT), University of Tübingen, Tübingen, Germany; Dartmouth College, United States of America

## Abstract

DNA methylation of CpG islands plays a crucial role in the regulation of gene expression. More than half of all human promoters contain CpG islands with a tissue-specific methylation pattern in differentiated cells. Still today, the whole process of how DNA methyltransferases determine which region should be methylated is not completely revealed. There are many hypotheses of which genomic features are correlated to the epigenome that have not yet been evaluated. Furthermore, many explorative approaches of measuring DNA methylation are limited to a subset of the genome and thus, cannot be employed, e.g., for genome-wide biomarker prediction methods. In this study, we evaluated the correlation of genetic, epigenetic and hypothesis-driven features to DNA methylation of CpG islands. To this end, various binary classifiers were trained and evaluated by cross-validation on a dataset comprising DNA methylation data for 190 CpG islands in HEPG2, HEK293, fibroblasts and leukocytes. We achieved an accuracy of up to 91% with an MCC of 0.8 using ten-fold cross-validation and ten repetitions. With these models, we extended the existing dataset to the whole genome and thus, predicted the methylation landscape for the given cell types. The method used for these predictions is also validated on another external whole-genome dataset. Our results reveal features correlated to DNA methylation and confirm or disprove various hypotheses of DNA methylation related features. This study confirms correlations between DNA methylation and histone modifications, DNA structure, DNA sequence, genomic attributes and CpG island properties. Furthermore, the method has been validated on a genome-wide dataset from the ENCODE consortium. The developed software, as well as the predicted datasets and a web-service to compare methylation states of CpG islands are available at http://www.cogsys.cs.uni-tuebingen.de/software/dna-methylation/.

## Introduction

DNA methylation of differentiated cells in mammals occurs almost exclusively at the C5 position in cytosine when it is immediately followed by a guanine [1]. The methylation of the 5′–CG–3′ pair is related to chromatin remodeling effects and mostly results in silencing of downstream genes [2], [3]. CpGs are significantly enriched in some parts of the genome, compared to the average CG content of the whole genome. These CpG enriched genome parts are called CpG islands [4]. CpG islands are usually detected computationally, e.g., by applying certain constraints on the occurrence of CpGs in a sequence window [5]. More recent approaches try to include further data and conditions to get better predictions of CpG islands in the genome [6]. Therefore, depending on the chosen approach and constraints, the number of CpG islands in the human genome varies. However, it is known that CpG islands occur in 50 to 60 percent of all human promoters and in all promoters of human housekeeping genes [7]–[9].

These CpG islands are mostly unmethylated and therefore represent a markable exception to the almost globally methylated genome [7], [10]. Today, it is known that different tissues and cell lines have specific methylation profiles [11]–[14]. These profiles are inherited by epigenetic mechanisms which are not completely understood [15]. Though, there are some recent evidences that DNA methylation profiles during early development of cells is probably mediated through histone modifications [10]. However, not only different tissues exhibit a specific methylation profile but also diverse diseases are suspected to have specific alterations of the usual methylation profile [16]. Especially in cancer, DNA methylation is supposed to play a key role for the repression of tumor suppressor genes [17]–[19]. Due to the large amount of different tumor types, cell lines and tissues with each having an own methylation profile, many explorative approaches to determine the DNA methylation status are required. A common way to experimentally perform DNA methylation analyses by hand is, to employ bisulfite sequencing and focus only on selected genomic regions [20], [21]. Prediction methods that extend data to the whole genome can be employed to reduce the experimental costs and speed up the methylation detection process [22]–[24]. But more important, they can be used to gain insights of the DNA methylation process. For example, they can reveal which features are of influence for a specific methylation pattern of a particular tissue or disease. These prediction methods need numeric features to distinguish between methylated and unmethylated CpG islands. The search for features to predict the methylation status of CpG islands started with Feltus *et al.* [25], who used general CpG island attributes (such as CG content, CG observed/expected ratio, etc.) and static sequence motifs as features. Das *et al.* [26] additionally introduced Alu coverage and general di- and trinucleotides as features. Fang *et al.* [27] extended the prediction of transcription factor binding sites from motifs to position frequency matrices and used them as features. So far, only purely sequence based features have been used. Bock *et al.* [28] were the first to introduce new kinds of features. In addition to the features from previous approaches, they used repeat frequencies and distributions, CpG island frequencies and distributions, predicted DNA structure, gene and exon distribution, evolutionary conservation, and SNPs. Later approaches did not introduce more relevant features to the prediction of CpG island methylation, except for Fan *et al.*, who successfully used histone modification marks for this task [29]. Apart from these methylation prediction approaches, several research groups suggested some distinctive features based on their studies. For example, the distance to the transcription start site, periodic distributions of CpGs in methylated CpG islands, flanking sequences of methylated CpGs, or tissue-independent correlation of histone modification profiles and DNA methylation [10], [20], [21], [30], [31].

In this study, we used data from the NAME21 consortium [21] to assess the accuracy of different prediction algorithms (see Table 1) and the predictive performance of different genomic and epigenomic features (see Table 2 for a complete list). For this purpose, we created and analyzed feature sets for four different cell types, consisting of nearly all features from previous approaches, new findings of recent studies, and completely new features. Afterwards, we extended the given data from chromosome 21 for HEPG2, HEK293, leukocytes and fibroblasts to a whole-genome catalogue of DNA methylation in human and developed a webservice that allows viewing the results and comparing them between the different cell types. Furthermore, we validated our method on a dataset from the ENCODE consortium [32] and released a stand-alone application for generating the described feature sets.

**Table 1:** Machine learning algorithm performance.

	HEPG2	HEK293	Leukocytes	Fibroblast
**LIBSVM (RBF kernel)**	0.543±0.30	0.716±0.01	0.801±0.02	0.635±0.19
**LIBSVM (linear kernel)**	0.382±0.24	0.743±0.17	0.825±0.14	0.564±0.18
**Random Decision Forest**	0.363±0.28	0.667±0.19	0.765±0.18	0.333±0.32
**kNN**	0.383±0.27	0.654±0.19	0.683±0.21	0.407±0.28
**Decision tree (J48)**	0.204±0.24	0.526±0.20	0.629±0.26	0.214±0.30
K*	0.128±0.33	0.381±0.36	0.393±0.36	0.312±0.40
**Naive Bayes**	0.057±0.21	0.117±0.26	0.146±0.22	0.064±0.24

Performance comparison of different machine learning algorithms for the task of DNA methylation prediction. We measured Matthews correlation coefficient (MCC) for every algorithm and every cell type using all features. The values shown in this table are the average of ten repetitions using ten-fold cross-validation.

## Results and Discussion

This study is based on data from Zhang *et al.*, who experimentally determined the methylation status of 190 CpG islands on chromosome 21 using 297 amplicons [21]. We extracted data for DNA methylation of CpG islands from this dataset for four cell types: leukocytes, fibroblasts, HEPG2 and HEK293. For each CpG island in each cell type, we calculated values for the following feature classes separately as described in the methods section: (1) distances to transcription start sites, (2) CpG island-specific attributes (e.g., CG content, CG ratio), (3) genomic attributes (e.g., number of surrounding exons, transcripts), (4) repeat, Alu-Y and DNA/DNA alignment features, (5) single nucleotide polymorphisms (SNPs), (6) periodic CpG distances, (7) closest CpGs, (8) sequence - dinucleotides, (9) sequence - tetranucleotides, (10) CpG flanking sequence, (11) splice sites, (12) transcription factor binding sites, (13) DNA structure, (14) evolutionary conservation, (15) histone modification data. Using the complete set of features, we applied the following machine learning algorithms to assess their performance on CpG island methylation prediction: (A) decision trees (J48), (B) naive Bayes, (C) k-nearest neighbor, (D) K* [33], (E) random decision forest, (F) and support vector machines with Gaussian radial basis function and (G) linear kernel. To ensure a fair comparison, all analyses have been repeated ten times with a ten-fold cross-validation. Model selections have been performed for each classifier, if required. Using the most accurate classifier, we evaluated the suitability of all 15 feature classes for predicting DNA methylation of CpG islands. To this end, we trained and evaluated support vector machines with RBF kernel for all 15 feature classes and four cell types separately and evaluated their performance. With these results in hand, we extended the existing methylation data to the whole genome, by predicting the unknown methylation status of all CpG islands in the human genome. To further evaluate the generalizability of our method, we took a whole-genome DNA methylation dataset from the ENCODE consortium [32] and trained our classifier on all CpG islands from chromosome 21. We achieved an accuracy of 90%, while predicting the remaining chromosomes and comparing the predicted to the experimental methylation state.

### Machine Learning Algorithms

Table 1 summarizes the performance of each machine learning algorithm. To ensure a fair comparison of different algorithms and kernels, model and parameter selections have been implemented for all algorithms, as required. All values are an average of ten repetitions of ten-fold cross-validations. Support vector machines (SVMs) outperformed other machine learning techniques in most datasets. We could not measure a significant difference between linear and radial basis function (RBF) kernels across all cell types. Since the explanatory power of the accuracy relies strongly on the underlying distribution (count of methylated and unmethylated CpG islands), we used Matthews correlation coefficient (MCC) for comparing the machine learning algorithms. The maximal correlation coefficient achieved is 0.825 when predicting the methylation status of leukocytes with a linear kernel. In general, SVMs outperformed other machine learning approaches in our comparison. However, we could not measure a significant difference between RBF and linear kernels across all cell types. The second best classifiers according to Table 1, are random decision forest and the k-nearest neighbor (kNN) algorithm. In essence, kNN is based on measuring the numerical distance of feature values to methylated or unmethylated instances and assigning the state of the closer instance. The good performance of this algorithm indicates that certain features must be correlated to DNA methylation. This again supports the assumption that there are DNA- and sequence-related features that increase the probability that cytosines are getting methylated. Furthermore, Table 1 shows that K*, and naive Bayes are not suitable for good DNA methylation predictions. Decision trees show a decent performance in HEK293 and Leukocytes, but fail to predict the other two cell types. Consequently, it is possible that they fail to accurately predict DNA methylation on unknown datasets.

### Features

**Figure 1:**
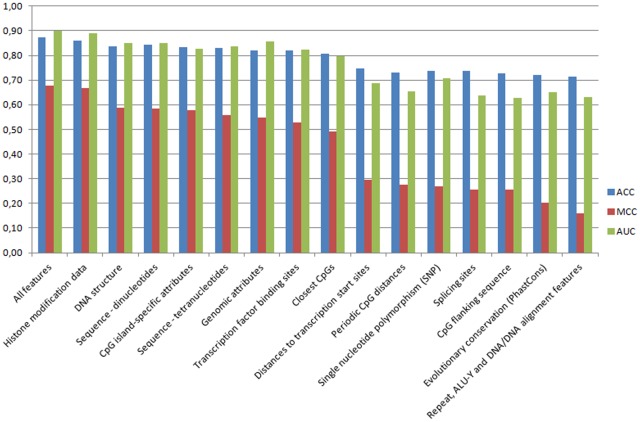
CpG island methylation predictions with individual feature classes reveal, which features are correlated to the epigenome. The figure shows the predictive performances of feature classes, averaged across HEPG2, HEK293, leukocytes and fibroblasts. It reveals, which features are correlated to DNA methylation and which are unlikely to be related to DNA methylation. Each value is an average of a ten-fold cross-validation with ten repetitions. The figure shows the accuracy (ACC), Matthews correlation coefficient (MCC) and the area under the receiver operating characteristic curve (AUC) and is sorted by average MCC.

To evaluate the predictive performance of our features and to analyze, which conditions make cytosines more prone to getting methylated than others, we divided the generated set of features. One feature set was created for every cell type and every feature class, resulting in 4.15 = 60 feature sets. We performed each experiment for every feature set using support vector machines with RBF kernel. The performance for each class of features in each cell type is shown in Table 2. Figure 1 gives an impression which genomic and non-genomic features are correlated to DNA methylation across all cell types. Taking the accuracy as a measure of performance is not recommended, because the underlying data is unbalanced. Thus, assigning all CpG islands the unmethylated state would result in an accuracy between 60% and 74%. A better measure is the MCC, since it takes both states (methylated and unmethylated) equally into account and is thus independent of the class distribution. An MCC of -1 denotes a perfect inverse prediction, whereas an MCC of 1 denotes a perfect prediction. An MCC of 0 corresponds to an average random prediction independent of the underlying class distribution. In this section, we are going to report and discuss all features in order of their predictive power. This is the same order as they appear in the referenced tables and figures.

**Table 2:** Single feature class performance.

Feature name	HEPG2		HEK293		Leukocytes		Fibroblast	
	ACC	MCC	ACC	MCC	ACC	MCC	ACC	MCC
All features	0.85	0.54	0.87	0.72	0.91	0.80	0.87	0.64
Histone modification data	0.83	0.52	0.83	0.66	0.91	0.82	0.86	0.68
DNA structure	0.82	0.47	0.85	0.68	0.85	0.67	0.83	0.53
Sequence - dinucleotides	0.80	0.34	0.86	0.70	0.89	0.76	0.82	0.54
CpG island-specific attributes	0.79	0.41	0.86	0.69	0.87	0.71	0.81	0.50
Sequence - tetranucleotides	0.78	0.34	0.86	0.69	0.89	0.75	0.79	0.45
Genomic attributes	0.82	0.47	0.83	0.63	0.82	0.60	0.81	0.49
Transcription factor binding sites	0.81	0.41	0.80	0.58	0.85	0.65	0.82	0.48
Closest CpGs	0.82	0.43	0.78	0.52	0.81	0.56	0.82	0.45
Distances to transcription start sites	0.76	0.19	0.72	0.40	0.73	0.36	0.77	0.24
Periodic CpG distances	0.76	0.26	0.67	0.27	0.73	0.38	0.76	0.20
Single nucleotide polymorphism (SNP)	0.77	0.23	0.67	0.27	0.71	0.31	0.78	0.27
Splicing sites	0.80	0.35	0.65	0.19	0.72	0.34	0.77	0.15
CpG flanking sequence	0.79	0.32	0.68	0.29	0.65	0.13	0.78	0.28
Evolutionary conservation (PhastCons)	0.78	0.16	0.65	0.26	0.68	0.21	0.77	0.18
Repeat, ALU-Y and DNA/DNA alignment features	0.76	0.11	0.65	0.23	0.68	0.22	0.77	0.08
Unmethylated instances [%]	0.74		0.60		0.65		0.74	

Comparison of predictive performances of single feature classes. All values are taken from SVM predictions with feature files that only contain features belonging to the given class. Each prediction is an average of a ten-fold cross-validation with ten repetitions. The table shows the accuracy (ACC) and Matthews correlation coefficient (MCC) for each cell type and each feature class and is sorted by average MCC. Please note that the underlying data is imbalanced (because CpG islands tend to be unmethylated) and the average accuracy when assigning all CpG islands the unmethylated state is 0.71.

The best average performance is achieved when using all features. This confirms a correlation of DNA methylation to features from different categories. Nearly the same performance is achieved when using exclusively the histone modification feature. This indicates that DNA methylation of CpG islands is strongly correlated to histone modifications as previously suggested by several other studies [29], [34], [35]. This result is also a confirmation of the proposed method, since recent studies have shown that the basic DNA methylation profile during early development is probably mediated through histone modifications [10], [36].

Before establishing the tissue-specific DNA methylation profile, some genomic regions are wrapped around nucleosomes that contain methylated lysine 4 of histone H3 (mono-, di- or trimethylation - H3K4me). Other nucleosomes contain unmethylated H3K4. This methylated H3K4me mark prevents *de novo* methylation of CpG islands in the embryo [10]. Since these findings are tissue-independent, other researchers have detected inverse relations between DNA methylation and H3K4me or H3K27me for multiple cell types [37], [38]. The histone modification dataset used in this study (that includes separate data for H3K27 and H3K4 mono-, di- and trimethylation) has been measured in human CD4 T-cells. The strong predictive performance of this feature class across all cell types supports these recent findings and confirms that H3K4me marks dictates methylation of CpG islands across several cell types. Another strong correlation exists between DNA structure and methylation. This has already been confirmed in other prediction approaches [28], [39]. Bock *et al.* report that the DNA rise (displacement between two base pairs along the helix axis) increases generally in CpG islands, compared to other genomic regions, while the DNA twist (amount of rotation between two base pairs around the helix axis) decreases. Methylated CpG islands seem to enhance this effect and have a much higher difference in DNA rise/twist than unmethylated CpG islands. Interestingly, this feature class performs in all different cell-types nearly as well as histone methylation marks.

Typical feature classes, that have already been used by the first CpG island methylation prediction approaches, are DNA sequence (di- or tetranucleotides) and CpG island specific attributes (such as CG content, CG observed/expected ratio, etc.) [25], [26]. They have been proven to be good features for discriminating between methylated and unmethylated CpG islands. Using just the dinucleotides, classification of methylated CpG islands in some cell types is even superior to the DNA structure feature class. This again confirms the strong correlation between DNA sequence and methylation. Genomic attributes and transcription factor binding sites also have a good correlation to DNA methylation. Genomic attributes include, e.g., the number of exons overlapping with the CpG island and it has been shown that exons tend to be higher methylated than introns [40], [41]. Thus, this is an evident feature for DNA methylation predictions. Using Transcription factor binding sites (TFBS) to predict DNA methylation of CpG islands has been introduced by Fang *et al.* [27]. We calculated the binding scores of 1539 potential transcription factors in all CpG islands of our input dataset. 456 of those had significant binding scores. Those have been taken to calculate the features and measure their correlation to DNA methylation. We observed that there are no single transcription factors with a high correlation to DNA methylation, whereas the entirety of all TFBS in this class has a good prediction score. TFBS are typically modeled by creating position frequency matrices for target sequences of a transcription factor. As a result, TFBS mainly depend on the underlying DNA sequence. Thus, having in mind that DNA sequence, in general, is a good discriminator for CpG island methylation, it is possible that the correlation between DNA methylation and TFBS is simply due to the underlying DNA sequence.

The periodic CpG distances features are based on hypotheses of Jia *et al.* and Zhang *et al.* [21], [30]. Both reported that if CpGs inside a CpG island occur at distances of approximately *x*.9 base pairs (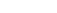), the CpG island tends to be methylated. Based on these findings, we added features reflecting this hypothesis. But our research shows that this hypothesis is not suitable to discriminate between methylated and unmethylated CpG islands. We further investigated this hypothesis by adding a novel, more generalized feature class, i.e., the average distance between CpGs in CpG islands. These features perform slightly better than considering only multiples of nine, but should still be combined with other feature classes to get overall good predictions. Another hypothesis-driven feature class is the noticeable hypomethylation when approaching transcription start sites (TSS). Eckhardt *et al.* reported an almost unmethylated core region of about ±1000 bps of each TSS [20] and Zhang *et al.* propose that CpG islands, overlapping a TSS, are mostly unmethylated in differentiated cells [21]. Our research shows that this feature is not generalizable. This means that a CpG island is more likely to be unmethylated when it is close to a TSS, but it does not mean anything if it is not. Probably just like this feature class, other feature classes only come into play in special conditions but are not eligible for predicting the methylation status of CpG islands genome-wide.

Furthermore, we investigated correlations between DNA methylation and single nucleotide polymorphisms (SNPs) and conclude that there is no general relation between those. The same holds true for correlations between DNA methylation and splicing sites. But, for SNPs, it might be possible to use them as specific predictors for DNA methylation, if the data is obtained from the same samples (see, e.g., Bell *et al.* [42]). Evolutionary conservation is a feature which seems to be correlated to DNA methylation, because CpG islands are evolutionarily conserved regions. This feature is probably more appropriate to detect CpG islands itself, but not for the methylation state. The so called *PhastCons*, which have previously been included by other groups [28], [39], did not perform well in our study. Another controversial hypothesis-driven feature is the flanking sequence preference of DNA methylating proteins. Several groups reported different flanking sequence preferences of CpGs that makes them more prone for getting methylated [21], [31], [43]. In our studies, trying to predict the DNA methylation status by flanking sequence preference does not lead to good results. However, this does not mean that there is no flanking sequence preference. For example, Oka *et al.* [44] experimentally confirmed the results by Handa *et al.* [31] and detected a flanking sequence preference of DNMT3A. Overall, one has to consider that *de novo* methylation of DNA is performed by the DNA methyltransferase enzymes DNMT3A and DNMT3B complexed with DNMT3L [10]. Comparing this low number of DNA methylating enzymes with the number of CpGs in the human genome, one can imagine that it is very hard to derive specific flanking sequences to discriminate between methylated and unmethylated CpGs. Because, even when trying to derive flanking sequences for every enzyme separately, one would have to divide the set of all CpGs in only three classes: methylated by DNMT3A, methylated by DNMT3B and unmethylated. Hence, our studies show that there is no general flanking sequence, which makes certain CpGs more prone to methylating proteins than others.

Table 2 shows that the absolute predictive performance of some feature classes varies between different cell types. This could be due to technical reasons, like the variability in the number of training instances between the different cell types, or the varying ratios between methylated and unmethylated CpG islands. It is also possible that these deviations come from biological reasons, e.g., in some cell types, different features are more correlated to DNA methylation than in others. But, Table 2 also shows that the relative predictive performance of all feature classes is fairly consistent for all cell types. Thus, in case of DNA methylation predictions for novel datasets, we recommend a union of the best performing feature classes: histone modification data is always recommended, but might sometimes not be available. The DNA structure feature set can be calculated from the sequence alone, same holds true for the dinucleotides, CpG island specific attributes and Closest CpGs. These attributes, together with the genomic attributes features, form a good set for novel predictions. All features sets below Closest CpGs (see Table 2) are not recommended, because they are too inaccurate. The tetranucleotides are redundant to the dinucleotides. The transcription factor binding sites might be included in novel feature sets, but they are slower and more difficult to calculate, compared to the other sequence-based features.

### Prediction of CpG Island Methylation Status

**Figure 2:**
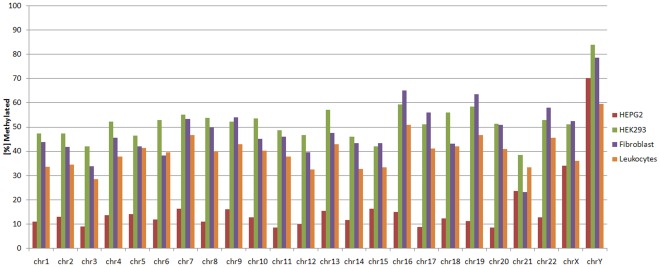
Predicted whole-genome methylation landscape for all four cell types. This figure visualizes the methylation landscape in all four cell types, compared to the total number of CpG islands. One bar represents the number of methylated instances per cell type as percentage of the total number of CpG islands in the given chromosome. The largest number of methylated CpG islands can be found in HEK293, whereas HEPG2 have an almost unmethylated genome. The few CpG islands in chromosome Y are hypermethylated in most cell types, compared to the other chromosomes.

We downloaded the coordinates of all CpG islands in the human genome from UCSC [45]. With the whole feature dataset for every cell type, we trained SVMs and took the best parameters to predict the methylation status of all CpG islands in the human genome. The methylation landscape of each cell type across the whole genome is visualized in Figure 2. We have set up a webservice at http://www.cogsys.cs.uni-tuebingen.de/software/dna-methylation/that allows users to select one or two cell types, a chromosome number and then view or compare the methylation status of CpG islands. This webservice includes all experimental data in the NAME21 and HEP datasets. Additionally, all predicted data for the cell types measured in the NAME21 data have been included. The webservice allows users to compare the CpG island methylation status of two cell types by distinguishing between CpG islands that are methylated, unmethylated and differentially methylated in both cell types. The data can be visualized using the UCSC genome browser [46]. An approximate score is generated for each prediction that represents the certainty of the prediction. In other words, this score represents the distance to the SVM hyperplane as per mille of the maximum predicted distance.

### Validation

To further assess the generalizability of our method (SVMs with RBF kernel, using all described features), we evaluated it on a whole-genome DNA methylation dataset from the ENCODE consortium [32]. Briefly, we mapped the probes to CpG islands (resulting in 17,588 instances), trained our classifier with cross-validation on a subset of the data and used the resulting model to predict the methylation state of the remaining CpG islands (more details can be found in the methods section). Afterwards, we counted the confusion matrix by comparing the experimentally determined methylation state with the predicted methylation state of all CpG islands that have not been used for the training. Depending on the size of the training and validation set, we achieved accuracies between 87.2% (trained on 10% of the data) and 92% (trained on 50% of the data), and MCCs from 0.48 (10%) to 0.58 (50%). Thus, predictive performance of the method increases with the size of data, available for training. To validate our NAME21 predictions, we further composed a training set, consisting of all CpG islands in chromosome 21 (resulting in 224 instances for which we had data in out validation dataset). Afterwards, we predicted with this model a validation set, consisting of all CpG islands in other chromosomes and compared the results to the experimentally determined methylation state. This resulted in an accuracy of 90% and an MCC of 0.43. Please see Table 3 for more detailed results on these validation runs.

**Table 3:** Validation on experimental data.

Trained on	TotalCGIs	CGIs in training set	CGIs in test set	ACC	MCC
CHR21	17588	224	17364	90.01	0.43
10%	17588	1758	15830	87.18	0.48
25%	17588	4397	13191	91.68	0.56
50%	17588	8794	8794	92.02	0.58

Validation of the proposed method (SVMs with RBF kernel, using all described features) on experimental data. The experimental dataset has been divided into a training and a test set. The training set was used for training and the test set exclusively for the comparison with prediction results and calculation of accuracy (ACC) and Matthews correlation coefficient (MCC). We performed this evaluation on four different training datasets: consisting of all CpG islands (CGIs) from chromosome 21, randomly picked 10%, 25% and 50% of the data.

### Evaluation of a Quantitative Prediction Approach

To explore the possibility of a quantitative DNA methylation prediction approach, we also employed support vector regression (SVR) models. Briefly, a SVR model is trained by using the actual methylation percentage of a CpG island instead of the binary distinction between methylated and unmethylated CpG islands. Consequently, these regression models return a methylation percentage, instead of a binary attribute. To assess the suitability of this classifier, we used the “GM12878 - replicate 1″ dataset from the ENCODE consortium [32] and processed it as described in [sec:mm:validation]the validation subsection of the methods section. We trained our classifier on 10% of the data and predicted the remaining 90%. For evaluating these quantitative models, error rates measuring the average difference between the actual and the predicted values are usually employed. We used the average absolute error (AAE) for performance evaluations (see Equation 1) and achieved an AAE of 0.117. This means that each predicted CpG island methylation value deviates on average by ±11.7%.

On the first glimpse, this is a good result. But regarding the fact that the genome-wide average methylation of our input dataset is only 9%, the SVR failed to successfully predict the few hypermethylated CpG islands. However, instead of predicting the actual amount of methylated CpGs in a CpG island, it is more useful to predict the binary methylation state of it. For example, if a CpG island is 80% methylated, it most likely suppresses gene expression, whereas it is very unlikely to suppress gene expression with 20% of all CpG’s being methylated. Thus, comparing the results of the SVR with our binary classifier validations, binary predictions of CpG island methylation are more accurate and lead to more meaningful results.

### Performance Comparison with Other Approaches

Comparing the performance of different methylation prediction approaches is difficult, because the procedure is usually a multi-step workflow (extracting CpG island coordinates, extending them, generating features, choosing a machine learning approach, performing the model selection, etc.) and there is no stand-alone application which can be requested from the authors of other published methods to make a fair comparison. Furthermore, some methods are tailored to a specific dataset and there is no standard operating procedure that can be used for performance comparison. Thus, the comparison has to be done by using the same input data and evaluation technique of those, who previously published methylation prediction results. Unfortunately, many datasets are not available anymore. We were unable to obtain a human brain dataset, used in Das *et al.* [26] and Fang *et al.* [27]. Further on, Bock *et al.* [28] and Fang *et al.* used data from the HEP pilot phase. Unfortunately, there is only data from the production phase of the HEP project available. Fan *et al.* [29] used a leave-one-out cross-validation statistic to validate their dataset, what makes their results hard to compare to others, because most other approaches are using a ten-fold cross-validation. However, the methylation prediction approach of Bock *et al.* is one of the latest approaches and probably the approach with highest impact. The CpG island coordinates of the training dataset and methylation states for human blood lymphocytes, used by Bock *et al.*, are publicly available. Furthermore, they also used support vector machines, which again makes their results well comparable to ours. Thus, we decided to make a comparison to Bock *et al.*, and add other approaches, who also published a comparison to Bock *et al.*, to our table.

We took the CpG island coordinates and the binary methylation state of the human blood lymphocytes dataset and lifted them to the NCBI 36 release of the human genome. Afterwards, we generated features and trained SVMs exactly as we did for the NAME21 data. The prediction results of our and other methods are shown in Table 4. Our maximal prediction accuracy on their dataset is 95.76% compared to 91.5% of Bock *et al.* and our maximum correlation coefficient is 0.87, compared to 0.74. This reflects the quality and suitability of the features used in our approach. For example, Bock *et al.* did not use the histone modification profiles, which are the best performing feature class in our approach. We also added the results of a comparison on the HEP pilot phase data to Table 4. Please note that comparisons on HEP data are popular but not recommended, because most amplicons they used do not fulfill CpG island criteria defined by Gardiner *et al.* [5]. This difficulty with the HEP data is also confirmed by Bock *et al.* and should be considered when comparing different approaches.

**Table 4:** Comparison of different methylation prediction approaches.

Year	Authors	Dataset	CC	Accuracy
2006	Fang *et al.* [27]	HEP pilot phase data	0.42	81.48
2006	Bock *et al.* [28]	HEP pilot phase data	0.15	74.76
2006	Bock *et al.* [28]	Human peripheral blood lymphocytes	0.74	91.5
*Our approach*		Human peripheral blood lymphocytes	0.87	95.76
*Our approach*		NAME21 (Leukocytes)	0.80	91.13

The predictive results of our method, compared to other methods. The table shows that our method outperforms other previously published methods.

### The Application

The Java application that has been developed to preprocess the input datasets and generate the features for this study is available at http://www.cogsys.cs.uni-tuebingen.de/software/dna-methylation/. This page also holds a documentation for the application, as well as example datasets, and the predicted methylation states for the NAME21 dataset. The application can read tab-separated files, containing probe or CpG island locations and methylation intensities. It can map probes to CpG islands, lift coordinates between different releases of the human genome and generate features for all 15 mentioned feature classes. The generated feature file can then be used with various machine learning applications (e.g., LIBSVM [47]) to train a model and evaluate classifiers or feature classes.

## Materials and Methods

### Datasets

This study is based mainly on a CpG island methylation dataset from the NAME21 Consortium. The dataset, published by Zhang *et al.*, is freely available [21]. Zhang *et al.* took the promoter regions of all protein coding genes on chromosome 21 in *Homo sapiens*, applied a window from 2000 bps upstream to 500 bps downstream of the transcription start site and searched for CpG enriched regions, using the Takai-Jones criteria [48]. These CpG islands have been analyzed in five different cell types: HEPG2 - a hepatocellular liver carcinoma cell line, trisomic fibroblasts - derived from an individual with Down syndrome, HEK293 - a human embryo kidney cell line, fibroblasts, and leukocytes. The methylation status of every cytosine has been determined using overlapping amplicons, in a way that most CpGs are covered by multiple amplicons, resulting in 297 amplicons for 190 genes. On the experimental side, they used bisulfite conversion and subclone sequencing to detect methylated CpGs [49]. We took their raw data and parsed it into a cell type specific data structure of CpG islands and CpGs. Methylation information from multiple amplicons for single CpGs have been averaged. To determine the methylation status of a CpG island, we averaged the methylation status of all CpGs in that CpG island and considered it methylated, if this value is above 60% (same threshold as in Bock *et al.* [28]).

After applying these constraints, our dataset consists of 56 methylated (112 unmethylated) instances for leukocytes, 73 methylated (117 unmethylated) instances for HEK293, 44 methylated (142 unmethylated) instances for HEPG2, 43 methylated (142 unmethylated) instances for fibroblasts, and 32 methylated (137 unmethylated) instances for trisomic fibroblasts. During evaluation of these datasets, we decided to remove the trisomic fibroblast dataset for this study, because it contains only of 32 methylated CpG islands with 81% of all CpG islands being unmethylated. The low number of training samples in this highly imbalanced dataset made it unsuitable for reliably train support vector machines with a ten-fold cross-validation.

We extended the sequence to analyze for each CpG island from the given coordinates by the primer sequence length and 500 bps up- and downstream to also cover nearby effects, which might have an influence on cytosine methylation. For example, *cis*-acting transcription factors might lie in the sequence, flanking the CpG island [25]. This window size has also been chosen by Fan *et al.* [29] and approved as a good choice by Fang *et al.* [27]. We double-checked the data by retrieving every single CpG island sequence from Ensembl and comparing it to the sequence given in the source data.

Furthermore, we used two datasets from the ENCODE consortium [32] to evaluate a quantitative DNA methylation prediction approach and to validate our method. For the quantitative DNA methylation prediction approach, we used the “ENCODE HudsonAlpha Methyl27 GM12878 replicate 1″ dataset [50] and to validate our method, the “ENCODE HudsonAlpha MethylSeq HEPG2, Pcr2x, replicate 1″ dataset [51] has been used.

### Machine Learning Algorithms and Scoring

In order to evaluate the predictability of our features, various machine learning algorithms have been considered. We used support vector machines (SVMs) with linear and radial basis function (RBF) kernels, decision trees, naive Bayesian networks, k-nearest neighbor, random decision forest and the K* (KStar) classifier.

With each of these machine learning algorithms, we assessed the predictive performance using the complete feature dataset for each cell type separately. The accuracy, Matthews correlation coefficient (MCC) and the area under the receiver operating characteristics curve (AUC) have been calculated for each prediction. The accuracy is the percentage of all predictions that are correct. The MCC is a performance measure that is especially suited for imbalanced binary datasets. It calculates a correlation coefficient between -1 (perfect inverse prediction) and 1 (perfect prediction), where 0 is an average random prediction independent of the underlying class distribution. This is a good measure for DNA methylation predictions because CpG islands tend to be unmethylated. For example, with 71% of all CpG islands being unmethylated, simply classifying all data as unmethylated would already result in an accuracy of 71% but the MCC would be 0. For a detailed discussion on these scoring metrics and their calculation, please see the work of Baldi *et al.* [52].

To measure the performance of support vector regression models, we employed the average absolute error (AAE), which is an error rate that measures the average difference between the actual (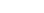) and the predicted (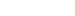) methylation values (see Equation 1).
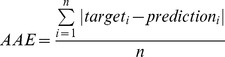
(1)


All experiments have been performed using a ten-fold cross-validation. In addition, ten repetitions with different seeds have been used in all experiments, resulting in 100 experiments, which were averaged for each reported value. For the SVM predictions (classification and regression), the LIBSVM [47] and LIBLINEAR [53] have been used. The WEKA library [54] has been used for all other classifiers.

### Validation

The HEPG2 dataset from ENCODE, used for the validation of our method, is a binary dataset. It contains sequence regions that have a score of 0 (unmethylated) or 1000 (methylated). All sequence regions from this dataset have been mapped to CpG islands (using coordinates from UCSC) and each CpG island is assigned a methylation value, based on the average methylation of all sequence regions, overlapping with the CpG island. Sequence regions that did not overlap with CpG islands have been discarded. Afterwards, we removed all differentially methylated CpG islands from our dataset. For example, if a CpG island has two overlapping sequence regions and only one of them is methylated, even with the experimental data we can not decide if the whole CpG island is methylated or not. Thus, we removed all CpG islands that are between 40% and 60% methylated. Afterwards, we separated this dataset into a training and a test set. This is performed, e.g., by randomly picking 10% of all CpG islands using a stratified sampling procedure. This means, our training set has the same percentage of methylated CpG islands as the whole dataset. For another validation, we simply took all CpG islands from chromosome 21 into our training set and declared all CpG islands from other chromosomes as test set. After splitting the dataset, we trained a support vector machine with RBF kernel, using all features, ten repetitions and a ten-fold cross-validation on this training set. With the resulting model, we predicted the methylation status of the remaining CpG islands and compared them with the experimentally determined methylation status. This ensures an accurate validation, because no CpG islands that have been used to train the method are used to evaluate the predictions.

We used a similar procedure for the evaluation of our quantitative prediction approach. The GM12878 dataset from ENCODE, which is a non-binary dataset, has been used for this purpose. We mapped all sequence regions from this dataset to CpG islands annotated by UCSC, averaged the methylation values of all overlapping regions and removed regions that are not overlapping with CpG islands. We then picked 10% of these CpG islands as in the HEPG2 ENCODE dataset and evaluated our regression model by comparing all predicted methylation values with the experimental methylation values.

### Features

If not explicitly stated, all data comes from Ensembl v47. This Ensembl version is based on the NCBI 36 release of the human genome. We used coordinates and sequences from the NCBI 36 release for all studies. For all UCSC data, we used the hg18 version (which corresponds to NCBI 36). All features were calculated on this release of the human genome. We used the UCSC LiftOver tool (http://genome.ucsc.edu/cgi-bin/hgLiftOver) to map all data on this release of the human genome. Our feature dataset consists of 948 features from 15 categories:

1. **Distances to transcription start sites (4 features).** Several groups observed that the methylation of CpGs decreases gradually when approaching the transcription start site (TSS) of a gene [20], [21]. We included this observation by measuring the distance to the closest gene and closest protein coding gene, based on the Ensembl database. Each value has been added normalized and logarithmized.
2. **CpG island-specific attributes (7 features).** CG content, CG ratio, CG observed/expected ratio [5], CG/TG ratio (with and without the reverse strand), AT/CG ratio and a boolean flag, if the CpG island is in a coding region have been added as features.
3. **Genomic attributes (11 features).** We included the percentage of repetitive base pairs (CpG island length/total length of all self-alignments), number of genes overlapping with the CpG island, total length of all overlapping genes, number of exons overlapping with the CpG island, total and average length of all overlapping exons, number of transcripts for all overlapping genes and number of transcripts divided by number of overlapping genes. For completeness: CpG island length, percentage of CpGs in the whole CpG island and average distance between CpGs have been added to this category.
4. **Repeat, Alu-Y and DNA/DNA alignment features (19 features).** We implemented features, representing DNA/DNA self alignments in the CpG island region, various features covering repetitive elements in the CpG island region (e.g., total number of repeats, length of repeats) and multiple features analyzing the Alu-Y repeat. This special *AGCT*-repeat has been found to occur often in methylated CpG islands [27], [55]. All features were calculated for three different window sizes: ±900 bp, ±400 bp and exactly covering the CpG island.
5. **Single nucleotide polymorphism (8 features).** Known single nucleotide polymorphisms (SNPs) have been retrieved from the Ensembl database for various window sizes, flanking the CpG island. The number of all SNPs and the distance to the closest SNP from the center of the CpG island have been added to the feature list. Additionally, the same values have been calculated especially for T/C SNPs, because of its special role in bisulfite sequencing [56], [57].
6. **Periodic CpG distances (15 features).** Jia *et al.* reported a correlation between DNA methylation and a periodic distribution of CpGs [30]. They state that if the CpGs are at a distance of eight to ten base pairs, they are more likely to be methylated. Zhang *et al.* validated these findings and reported a significant enrichment of distances 9, 18 and 27 bps between CpGs in highly methylated amplicons [21]. Based on these findings, we calculated distance scores specially for multiples of nine from 9 to 45 bps in both directions of a CpG. While studying the flanking sequences of methylated CpGs, we realized a significant difference in CpG occurrence at a distance of 48 bps (CpGs occur almost twice as frequently as on other positions). For this reason, we added features representing the CpG occurrence at a distance of 48 bp on both strands, additionally to the multiples of nine. All values have been averaged for all CpGs in the CpG island and three additional features have been added, which represent sums of the multiples of nine, of the two 48 bps features and a sum of these two sums.
7. **Closest CpGs (6 features).** In addition to periodic CpG distances, feature scores have been implemented which measure the distance to the three closest CpGs in general. The features are calculated by measuring the distances to the three closest CpGs for all CpGs in a CpG island. The three smallest and the average of all values are added as features.
8. **Sequence - dinucleotides (16 features).** We counted the occurrences of all possible 16 dinucleotides in the CpG island sequence and added these values, divided by the CpG island length as features.
9. **Sequence - tetranucleotides (257 features).** We did the same for all 256 tetranucleotides. This also covers the four base pair long Alu repeat (for which we also added an additional feature that represents the total, not averaged count of Alu repeats). Please note that combining this feature class with the dinucleotides feature class might lead to redundancies in the resulting feature set.
10. **CpG flanking sequence (4 features).** Several authors claim to have found flanking sequences for DNA methyltransferases [21], [31], [43]. This means that methylated CpGs have other flanking sequences than unmethylated CpGs. Zhang *et al.* report that A/T nucleotides tend to occur more often in flanking sequences of methylated CpGs, whereas C/G flanks occur more often when the CpG pair is unmethylated [21]. We took the flanking sequences ±4 bp and ±20 bp for all CpGs in the dataset. We separated these into methylated and unmethylated CpGs and calculated position frequency matrices (PFMs) [58] for all these sequences and for every cell type separately as follows: Calculate a PFM for all methylated instances and a PFM for all unmethylated instances. Divide the PFM of methylated instances (by dividing the frequency of each nucleotide in each position) by the PFM of unmethylated instances. This leads to a total of four PFMs, separating methylated from unmethylated instances that were used for each cell type: two PFMs, specific for the cell type, with flanking sequence sizes of 4 and 20 bp and two non-specific PFMs with the same flanking sequence sizes. These PFMs have then been applied to the CpG island sequences and a weight score, covering the quality of the match and the significance, based on the frequency of the actual sequence in the whole human genome [59] is used as a feature. We used ModuleMaster [60] to apply the PFMs to the sequences and calculate the weight scores.
11. **Splice sites (5 features).** We used all four PFMs, generated from SpliceDB [61] to identify splice sites. The four PFMs correspond to mammalian frequency matrices of splice sites for GT-AG and GC-AG pairs for donor and acceptor sites respectively. These four features were integrated as weight scores (as described in *10) CpG flanking sequence*). Additionally, the number of hits from all PFMs has been added as fifth feature.
12. **Transcription factor binding sites (457 features** *).* Correlation between DNA methylation and transcription factor binding sites (TFBSs) has already been reported by several groups [20], [26]–[28]. ModuleMaster [60] was applied to calculate weight scores, as described in *10) CpG flanking sequence*, which we added to our feature database. The transcription factors have been selected among a large PFM database, consisting of transcription factor binding information from TRANSFAC professional, NUBIScan and predicted TFBSs. This dataset is described in more detail by Wrzodek *et al.* [60]. We took all CpG island sequences from the NAME21 dataset and performed a matrix scan with all PFMs on those. All PFMs which had a weight score below one were removed, because of lack of significance (good matches should have weight scores of at least five. Smaller scores indicate that either the putative TFBS is not well recognized by the PFM or that the putative TFBS occurs very often by chance throughout the human genome). This resulted in a total of 456 PFMs we took for this study. In addition to these 456 features, we added the logarithm of the sum of all TFBSs as feature.
13. **DNA structure (43 features).** Bock *et al.* have found a statistically significant correlation between DNA structure and CpG island methylation [28]. We used the data from Gardiner *et al.* to calculate octamer sequence dependent predicted DNA structure energies [62] (7th order hidden Markov models) and added a total of 43 features, representing these energy values.
14. **Evolutionary conservation (4 features).** Methylation patterns tend to be evolutionarily conserved [63]. Siepel *et al.* generated *PhastCon* elements, representing the evolutionary conversation of a region [64]. We took the data from the UCSC Genome Browser [45] and generated several features, representing the evolutionary conservation of the CpG island.
15. **Histone modification data (92 features).** The correlation between histone modification and DNA methylation has already been reported by several authors [10], [36], [65], [66]. Fan *et al.* already tried to use histone modification marks for predicting DNA methylation [67] and were quite successful with it. Barski *et al.* [29] generated 23 genome-wide datasets, covering 20 different histone modification variants (H3K4me1, H3K4me2, H3K4me3, H3K9me1, H3K9me2, H3K9me3, H3K27me1, H3K27me2, H3K27me3, H3K36me1, H3K36me3, H3K79me1, H3K79me2, H3K79me3, H3R2me1, H3R2me2, H4K20me1, H4K20me3, H4R3me2, H2BK5me1) and the distribution of H2A.Z, RNA polymerase II, and the insulator binding protein CTCF. We used this data, mapped it to each available CpG island and generated four numerical features for each histone modification dataset and CpG island. This results in a total of 92 histone modification features per CpG island. It has been shown that, e.g., H3K4me prevents DNA methyltransferase enzymes from *de novo* methylating CpG islands [10]. Thus, taking one dataset for all cell types allows for validation of our method, because some histone modifications dictate DNA methylation in the embryo.

### Prediction of CpG Island Methylation Status

All CpG islands have been downloaded for the hg18 release of the human genome from UCSC [45]. We generated feature sets for every CpG island exactly as we did for our training data. The LIBSVM *svm-predict* application [47] has been used with all features to predict the unknown methylation states. Two parameters (C and Gamma) for the RBF Kernel are necessary to build a model for the predictions. These have been estimated for each cell type by performing a grid parameter search on the experimental data and calculating the ACC, MCC and AUC of every parameter combination (parameter grids: C: 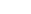 to 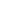 Gamma: 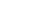 to 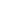 for each step, the exponent has been increased by 2). The grid has been extended if a maximum ACC, MCC or AUC lies on a border and refined to a smaller step size if a *peak* has been found. We took the parameter combination for the prediction that had the lowest combined score according to equation 2. In all cases, this score matched the maximal MCC.
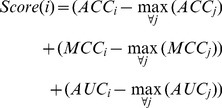
(2)


## References

[1] Bird AP (1978). Use of restriction enzymes to study eukaryotic DNA methylation: II. the symmetry of methylated sites supports semi-conservative copying of the methylation pattern.. Journal of Molecular Biology.

[2] Jones PA, Baylin SB (2007). The epigenomics of cancer.. Cell.

[3] Bernstein BE, Meissner A, Lander ES (2007). The Mammalian Epigenome.. Cell.

[4] Lander ES, Linton LM, Birren B, Nusbaum C, Zody MC (2001). Initial sequencing and analysis of the human genome.. Nature.

[5] Gardiner-Garden M, Frommer M (1987). CpG islands in vertebrate genomes.. Journal of Molecular Biology.

[6] Bock C, Walter J, Paulsen M, Lengauer T (2007). CpG Island Mapping by Epigenome Prediction..

[7] Antequera F, Bird A (1993). Number of CpG islands and genes in human and mouse.. PNAS.

[8] Antequera F (2003). Structure, function and evolution of CpG island promoters.. Cell Mol Life Sci.

[9] Wang Y, Leung FCC (2004). An evaluation of new criteria for CpG islands in the human genome as gene markers.. Bioinformatics.

[10] Cedar H, Bergman Y (2009). Linking DNA methylation and histone modification: patterns and paradigms.. Nat Rev Genet.

[11] Rollins RA, Haghighi F, Edwards JR, Das R, Zhang MQ (2006). Large-scale structure of genomic methylation patterns.. Genome Research.

[12] Schilling E, Rehli M (2007). Global, comparative analysis of tissue-specific promoter CpG methylation.. Genomics.

[13] Bustos CD, Ramos E, Young JM, Tran RK, Menzel U (2009). Tissue-specific variation in DNA methylation levels along human chromosome 1.. Epigenetics Chromatin.

[14] Brena RM, Huang THM, Plass C (2006). Toward a human epigenome.. Nat Genet.

[15] Reik W (2007). Stability and exibility of epigenetic gene regulation in mammalian development.. Nature.

[16] Costello JF, Frühwald MC, Smiraglia DJ, Rush LJ, Robertson GP (2000). Aberrant CpGisland methylation has non-random and tumour-type-specific patterns.. Nat Genet.

[17] Esteller M (2008). Epigenetics in cancer.. The New England Journal of Medicine.

[18] Brena RM, Costello JF (2007). Genome-epigenome interactions in cancer.. Human molecular genetics 16 Spec No.

[19] Esteller M (2007). Cancer epigenomics: DNA methylomes and histone-modification maps.. Nature Reviews Genetics.

[20] Eckhardt F, Lewin J, Cortese R, Rakyan VK, Attwood J (2006). DNA methylation profiling of human chromosomes 6, 20 and 22.. Nat Genet.

[21] Zhang Y, Rohde C, Tierling S, Jurkowski TP, Bock C (2009). DNA Methylation Analysis of Chromosome 21 Gene Promoters at Single Base Pair and Single Allele Resolution.. PLoS Genet.

[22] Zilberman D, Henikoff S (2007). Genome-wide analysis of DNA methylation patterns.. Development.

[23] Dehan P, Kustermans G, Guenin S, Horion J, Boniver J (2009). DNA methylation and cancer diagnosis: new methods and applications.. Expert Review of Molecular Diagnostics.

[24] Thu KL, Pikor LA, Kennett JY, Alvarez CE, Lam WL (2010). Methylation analysis by DNA immunoprecipitation.. Journal of Cellular Physiology.

[25] Feltus FA, Lee EK, Costello JF, Plass C, Vertino PM (2003). Predicting aberrant CpG island methylation.. Proceedings of the National Academy of Sciences.

[26] Das R, Dimitrova N, Xuan Z, Rollins RA, Haghighi F (2006). Computational prediction of methylation status in human genomic sequences.. Proc Natl Acad Sci U S A.

[27] Fang F, Fan S, Zhang X, Zhang MQ (2006). Predicting methylation status of CpG islands in the human brain.. Bioinformatics.

[28] Bock C, Paulsen M, Tierling S, Mikeska T, Lengauer T (2006). CpG Island Methylation in Human Lymphocytes Is Highly Correlated with DNA Sequence, Repeats, and Predicted DNA Structure..

[29] Fan S, Zhang MQ, Zhang X (2008). Histone methylation marks play important roles in predicting the methylation status of cpg islands.. Biochemical and Biophysical Research Communications.

[30] Jia D, Jurkowska RZ, Zhang X, Jeltsch A, Cheng X (2007). Structure of Dnmt3a bound to Dnmt3L suggests a model for de novo DNA methylation.. Nature.

[31] Vikas H, Albert J (2005). Profound Flanking Sequence Preference of Dnmt3a and Dnmt3b Mammalian DNA Methyltransferases Shape the Human Epigenome.. Journal of Molecular Biology.

[32] Celniker SE, Dillon LAL, Gerstein MB, Gunsalus KC, Henikoff S (2009). Unlocking the secrets of the genome.. Nature.

[33] Cleary JG, Trigg LE (1995). K*: An Instance-based Learner Using an Entropic Distance Measure. In: In Proceedings of the 12th International Conference on Machine Learning.. Morgan Kaufmann,.

[34] Henckel A, Nakabayashi K, Sanz LA, Feil R, Hata K (2009). Histone methylation is mechanistically linked to DNA methylation at imprinting control regions in mammals.. Hum Mol Genet.

[35] Fuks F (2005). DNA methylation and histone modifications: teaming up to silence genes.. Curr Opin Genet Dev.

[36] Ooi SKT, Qiu C, Bernstein E, Li K, Jia D (2007). DNMT3L connects unmethylated lysine 4 of histone H3 to de novo methylation of DNA.. Nature.

[37] Mohn F, Weber M, Rebhan M, Roloff TC, Richter J (2008). Lineage-specific polycomb targets and de novo DNA methylation define restriction and potential of neuronal progenitors.. Mol Cell.

[38] Meissner A, Mikkelsen TS, Gu H, Wernig M, Hanna J (2008). Genome-scale DNA methylation maps of pluripotent and differentiated cells.. Nature.

[39] Previti C, Harari O, Zwir I, del Val C (2009). Profile analysis and prediction of tissue-specific CpG island methylation classes.. BMC Bioinformatics.

[40] Jeltsch A (2010). Phylogeny of methylomes.. Science.

[41] Lister R, Pelizzola M, Dowen RH, Hawkins RD, Hon G (2009). Human DNA methylomes at base resolution show widespread epigenomic differences.. Nature.

[42] Bell JT, Pai AA, Pickrell JK, Gaffney DJ, Pique-Regi R (2011). Dna methylation patterns associate with genetic and gene expression variation in hapmap cell lines.. Genome Biol.

[43] Kim S, Li M, Paik H, Nephew K, Shi H (2008). Predicting DNA methylation susceptibility using CpG anking sequences..

[44] Oka M, Rodić N, Graddy J, Chang LJ, Terada N (2006). CpG sites preferentially methylated by Dnmt3a in vivo.. J Biol Chem.

[45] Rhead B, Karolchik D, Kuhn RM, Hinrichs AS, Zweig AS (2010). The UCSC Genome Browser database: update 2010.. Nucleic Acids Res.

[46] Kent WJ, Sugnet CW, Furey TS, Roskin KM, Pringle TH (2002). The human genome browser at UCSC.. Genome Res.

[47] Chang CC, Lin CJ (2001). LIBSVM: a library for support vector machines.. http://www.csie.ntu.edu.tw/~cjlin/libsvm.

[48] Takai D, Jones PA (2002). Comprehensive analysis of CpG islands in human chromosomes 21 and 22.. Proc Natl Acad Sci U S A.

[49] Schones DE, Zhao K (2008). Genome-wide approaches to studying chromatin modifications.. Nat Rev Genet.

[50] ENCODE HudsonAlpha Methyl27 GM12878 replicate 1. Downloaded from the “ENCODE Data Coordination Center at UCSC”.. http://hgdownload.cse.ucsc.edu/goldenPath/hg18/encodeDCC/wgEncodeHudsonalphaMethyl27/wgEncodeHudsonalphaMethyl27GM12878r1.bed.

[51] ENCODE HudsonAlpha MethylSeq HEPG2, Pcr2x, replicate 1. Downloaded from the “ENCODE Data Coordination Center at UCSC”.. http://hgdownload.cse.ucsc.edu/goldenPath/hg18/encodeDCC/wgEncodeHudsonalphaMethylSeq/wgEncodeHudsonalphaMethylSeqRegionsRep1Hepg2Pcr2x.bed9.gz.

[52] Baldi P, Brunak S, Chauvin Y, Andersen CA, Nielsen H (2000). Assessing the accuracy of prediction algorithms for classification: an overview.. Bioinformatics.

[53] Fan R, Chang K, Hsieh C, Wang X, Lin C (2008). LIBLINEAR: A library for large linear classification.. The Journal of Machine Learning Research.

[54] Hall M, Frank E, Holmes G, Pfahringer B, Reutemann P (2009). The WEKA data mining software: An update.. ACM SIGKDD Explorations Newsletter.

[55] Kochanek S, Renz D, Doerer W (1993). DNA methylation in the Alu sequences of diploid and haploid primary human cells.. EMBO J.

[56] Hajkova P, el Maarri O, Engemann S, Oswald J, Olek A (2002). DNA-methylation analysis by the bisulfite-assisted genomic sequencing method.. Methods Mol Biol.

[57] Bock C, Lengauer T (2008). Computational epigenetics.. Bioinformatics.

[58] Stormo GD (2000). DNA binding sites: representation and discovery.. Bioinformatics.

[59] Aerts S, Loo PV, Thijs G, Moreau Y, Moor BD (2003). Computational detection of cis-regulatory modules.. Bioinformatics.

[60] Wrzodek C, Schröder A, Dräger A, Wanke D, Berendzen KW (2010). ModuleMaster: A new tool to decipher transcriptional regulatory networks.. Biosystems.

[61] Burset M, Seledtsov IA, Solovyev VV (2001). Splicedb: database of canonical and non-canonical mammalian splice sites.. Nucleic Acids Res.

[62] Gardiner EJ, Hunter CA, Packer MJ, Palmer DS, Willett P (2003). Sequence-dependent DNA structure: a database of octamer structural parameters.. J Mol Biol.

[63] Bernstein BE, Kamal M, Lindblad-Toh K, Bekiranov S, Bailey DK (2005). Genomic maps and comparative analysis of histone modifications in human and mouse.. Cell.

[64] Siepel A, Bejerano G, Pedersen JS, Hinrichs AS, Hou M (2005). Evolutionarily conserved elements in vertebrate, insect, worm, and yeast genomes.. Genome Res.

[65] Ting AH, McGarvey KM, Baylin SB (2006). The cancer epigenome–components and functional correlates.. Genes Dev.

[66] Bird A (2002). DNA methylation patterns and epigenetic memory.. Genes Dev.

[67] Barski A, Cuddapah S, Cui K, Roh TY, Schones DE (2007). High-resolution profiling of histone methylations in the human genome.. Cell.

